# Pretreatment Multi-sequence Contrast-Enhanced MRI to Predict Response to Immunotherapy in Unresectable Hepatocellular Carcinoma Using Transformer: A Multicenter Study

**DOI:** 10.7150/jca.111026

**Published:** 2025-06-12

**Authors:** Jialin Chen, Juan Chen, Yamei Ye, Linbin Lu, Xinying Guo, Simiao Gao, Lifang Liu, Hongyi Yang, Chun Lin, Xiong Chen

**Affiliations:** 1 Department of Oncology, Fuzhou General Teaching Hospital of Fujian University of Traditional Chinese Medicine, 350001, Fuzhou, Fujian, PR China.; 2 Department of Oncology, Mengchao Hepatobiliary Hospital of Fujian Medical University, 350028, Fuzhou, Fujian, PR China.; 3 Department of Hepatology, Mengchao Hepatobiliary Hospital of Fujian Medical University, 350028, Fuzhou, Fujian, PR China.; 4 Department of Oncology, the 900th Hospital of Joint Logistic Support Force, PLA, Fuzong Clinical College of Fujian Medical University, 350001, Fuzhou, Fujian, PR China.; 5 Department of Oncology, Oriental Hospital Affiliated to Xiamen University, 350028, Fuzhou, Fujian, PR China.

**Keywords:** hepatocellular carcinoma, immunotherapy, MRI, predictive model, deep learning

## Abstract

***Background:*** Targeted combined immunotherapy (TCI) has shown certain antitumor effects in patients with unresectable hepatocellular carcinoma(uHCC), but only a subset of patients benefit. This study aims to develop a Transformer-based radiomics model to predict the objective response to combined therapy in patients with uHCC.

***Methods:*** This multicenter, retrospective study involved 264 HCC patients who underwent contrast-enhanced MRI prior to immunotherapy. The patients were divided into a training cohort(n=180) and a validation cohort(n=84). Using a multi-instance learning approach, tumor lesions in multi-sequence MRI were segmented into cross-sectional images, and features were extracted using the ResNet50 model. The Transformer model was then trained to predict the objective response rate (ORR). The prediction process was visualized using Grad-CAM and SHAP algorithms. Model performance was assessed using ROC and DCA curves, while survival analysis was conducted using Kaplan-Meier curves.

***Results:*** Among 264 patients, one achieved complete response (0.4%), 64 experienced partial response (24.2%). The ORR was 26.1% in the training group and 21.4% in the validation group. The model demonstrated high predictive accuracy, achieving a perfect area under the curve (AUC) of 1.000. Further validation using screenshot-based model inputs revealed an AUC of 0.929 (95% CI: 0.904, 0.947), confirming the model's clinical applicability. Kaplan-Meier analysis indicated that objective responders experienced better overall survival (OS) in both the training set (HR: 0.50, 95% CI: 0.27, 0.90) and the validation set (HR: 0.28, 95% CI: 0.08, 0.91).

***Conclusion:*** The deep learning framework combining ResNet50 and Transformer has proven its clinical applicability in predicting and assessing the efficacy of targeted combination immunotherapy in unresectable hepatocellular carcinoma, providing crucial guidance for clinical treatment decisions.

## Introduction

Hepatocellular carcinoma (HCC) presents a global health challenge, with projected increases in new cases and mortality rates exceeding 55% by 2040 1-3. In the realm of unresectable HCC (uHCC), recent years have seen significant advancements in treatment approaches, marked by the use of tyrosine kinase inhibitors (TKIs) 4 and immune checkpoint inhibitors (ICIs) 5. Notably, Nivolumab 6 and Pembrolizumab 7 as second-line treatment options have demonstrated substantial survival benefits, with overall response rates of 20% and 17%, respectively. In 2020, following the results of the IMbrave150 trial 8, the Food and Drug Administration (FDA) approved a combination of Atezolizumab and Bevacizumab as a new standard for first-line treatment 9, improving objective response rates to 27%. However, 70% of patients still fail to respond to these therapies (targeted combined immunotherapy, TCIs), resulting in significant losses in medical resources and financial cost. Consequently, there is an urgent need for new biomarkers to predict the effectiveness of TCIs, thus enabling personalized treatment plans for patients.

Current research indicates that artificial intelligence technologies, exemplified by radiomics 10, hold significant potential in the diagnosis 11, classification 12, and prognostic marker identification 13,14 of solid tumors. Wang et al. 15 developed an MRI-based radiomics model to predict the 5-year survival rate of HCC patients. Zhang et al. 16 combined deep learning features with clinical characteristics to develop a nomogram predicting individual prognosis for HCC patients undergoing TACE and Sorafenib treatments. Recent studies by Bo et al. 17 have also explored the feasibility of radiomics models in predicting responses to Lenvatinib treatment in patients with unresectable liver cancer. Despite these advancements, current research is often limited to single MRI sequence maximum regions of interest (ROI) features and does not fully utilize multidimensional MRI data 18 to capture comprehensive lesion information.

The Transformer model, one of the most popular deep learning architectures, effectively integrates multidimensional medical image data through its self-attention mechanism, offering breakthroughs in tumor analysis. In various medical imaging tasks such as MRI tumor segmentation 19 and pathological image cancer classification 20, Transformers have demonstrated exceptional capabilities. However, the potential of utilizing Transformer models to predict the response of uHCC patients to TCI treatments using multidimensional MRI data remains underexplored.

This study aims to propose a multidimensional MRI-based radiomics model to predict the response of uHCC patients to TCI treatment, thereby supporting clinicians in selecting the most appropriate treatment options.

## Methods

### Study Design and Patients

This retrospective multicenter study included 378 patients who were clinically or pathologically diagnosed with HCC between September 2017 and January 2023. These patients were recruited from four tertiary hospitals in China: Mengchao Hepatobiliary Hospital (Institution I), Xiangya Hospital (Institution II), Fujian Provincial Hospital (Institution III), and the 900th Hospital of PLA (Institution IV). All patients underwent Gd-EOB-DTPA-enhanced MRI examinations within two weeks prior to receiving combination therapy. The study was approved by the Ethics Committee of Mengchao Hepatobiliary Hospital (approval number: 2023_143_01), and the requirement for informed consent was waived.

### Clinical Data Collection and Follow-up

Comprehensive clinical data were obtained from electronic medical records, and contrast-enhanced magnetic resonance images (CE-MRI) in DICOM format were retrieved from the PACS system. In line with real-world clinical settings, patients were eligible to receive various anti-PD-1 antibody therapies. Tumor response was assessed bi-monthly (±2 weeks) following targeted combination immunotherapy (TCI) using CT or MRI. The responses were classified based on the RECIST v1.1 21 criteria into complete response (CR), partial response (PR), stable disease (SD), and progressive disease (PD). CR and PR conditions were required to be sustained for a minimum duration of two weeks. The follow-up period for this study concluded on November 15, 2023.

### CE-MRI Acquisition and Image Pre-processing

All patients underwent T2-weighted imaging (T2WI)-enhanced MRI scans using a Siemens Verio 3.0T superconducting MRI scanner equipped with an 8-channel body coil. The imaging protocol included multiple phases: pre-contrast (PRE), arterial phase (AP), portal venous phase (PVP), and delayed post-contrast phase (DP). Prior to feature extraction, the images were processed with grayscale discretization and resampling to standardize data across different institutions and scanners. The resampling parameters were set at 1 mm×1 mm×1 mm, and Hounsfield units were discretized into 25 bins to address potential variations in imaging acquisition.

### Regions of Interest Segmentation

Two experienced oncologists manually delineated the tumor boundaries layer-by-layer using ITK-SNAP software (version 3.8.0; http://www.itksnap.org/pmwiki/pmwiki.php) on axial T2-weighted images (T2WI) and sagittal 3D contrast-enhanced T1-weighted images (3D-CET1WI). The largest lesion in each layer was identified and saved as the ROI. In cases where there were disagreements regarding the ROI, a senior oncology expert provided the final decision. For patients with multiple tumors, the largest visible lesion on the MR images was selected as the ROI, given that multiple tumors generally indicate a higher tumor burden.

### 2.5D Segmentation Methods and Feature Extraction

The framework for developing and validating the predictive model is illustrated in Fig. 2. To balance parameter efficiency and preserve spatial heterogeneity in 3D convolutional neural networks, an improved 2.5D feature extraction strategy was implemented. Specifically, for 3D MRI data, the tumor's largest cross-sectional layer is first located in the sagittal plane. A ROI is then extracted within a ±4 voxel layer range along the longitudinal axis, and the 2.5D image data volume is constructed by stacking along the channel dimension to generate multi-channel input data. Feature extraction is performed using a pre-trained ResNet50 network, where the convolutional layers capture local texture features, and the 1024×N-dimensional feature vector output from the global average pooling (GAP) layer serves as the high-level representation. Z-score normalization is applied using statistics from the entire dataset.

### Model Construction, Validation, and Visualization

Multi-phase MRI sequences (PRE, AP, PVP, DP) were processed as independent instances, forming a 1024×N dataset (where N represents the number of instances). Feature training and efficacy prediction were conducted using an 8-head attention Transformer model^22^. The model was employed for parallel computation to capture inter-phase feature correlations and dynamically generate an attention weight matrix to adaptively select key imaging biomarkers. Initially, the data passed through a linear layer to reduce the dimensionality to 512×N, then passes through two layers of self-attention neural networks, followed by a multilayer perceptron (MLP) layer that outputs patient-level prediction labels and linear prediction values followed by two layers of self-attention neural networks. The training process employs the AdamW optimizer (learning rate = 1×10^-5^, weight decay = 1×10^-5^), with a maximum training period of 50 epochs. Early stopping is applied, halting training if the loss function does not change for five consecutive epochs. To enhance clinical interpretability, Grad-CAM is integrated to generate class activation heatmaps for localizing spatially sensitive regions. Additionally, SHAP value back-projection is used to map the contribution of higher-order features back to the original image space.

### Statistical Analysis

Categorical variables were analyzed using the Chi-square test or Fisher's exact test, while continuous variables were compared using the Mann-Whitney U test and the Kruskal-Wallis test. A significance level of *P* <0.05 was applied. Model performance was evaluated through area under the curve (AUC) , with net benefits assessed using Decision Curve Analysis (DCA). Overall survival (OS) curves were plotted using the Kaplan-Meier method. All statistical analyses were conducted using R software (version 4.3.1) and Python (version 3.9).

## Results

### Patient Characteristics

**Figure 1:**
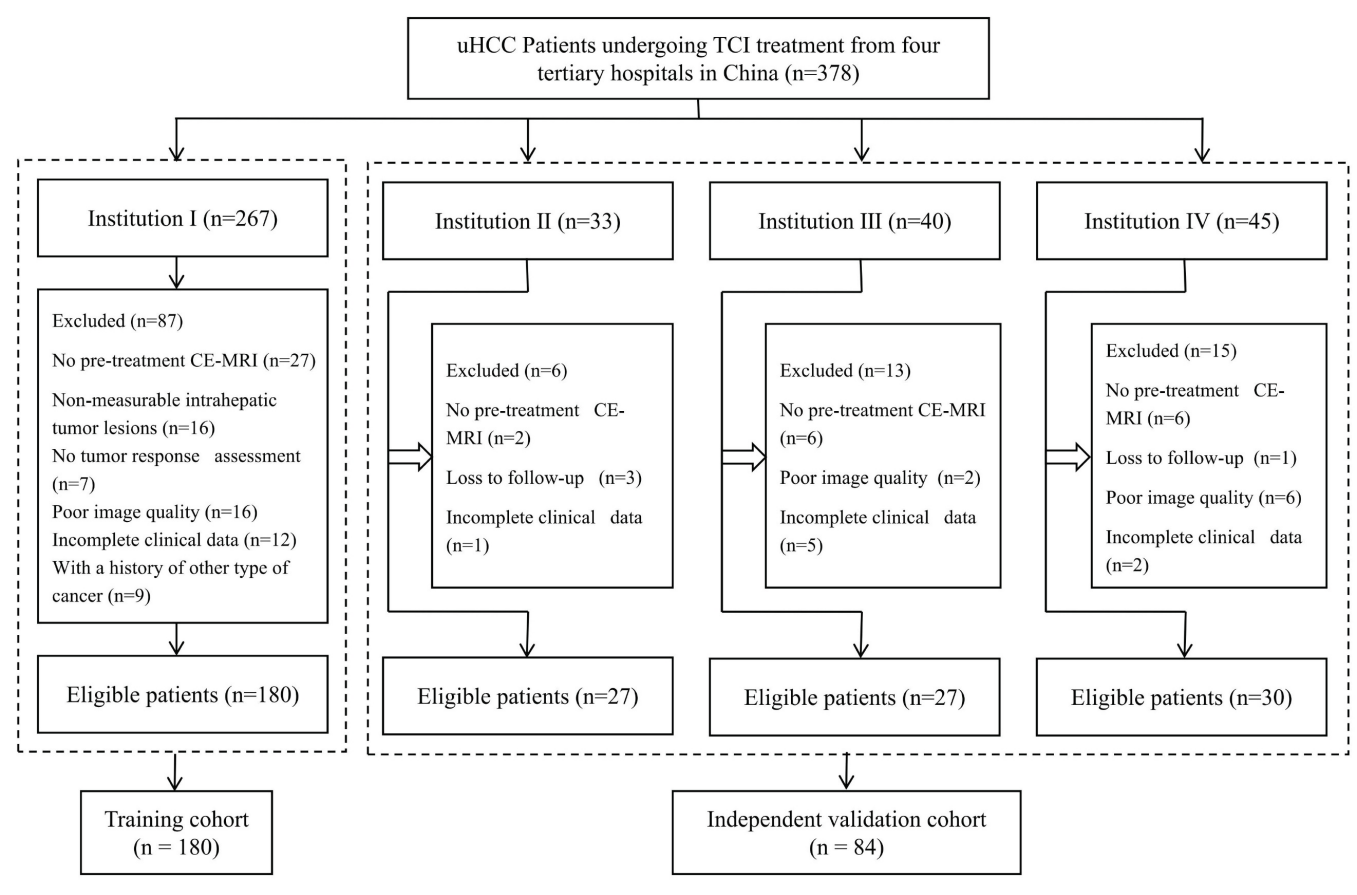
Flowchart of inclusion and exclusion criteria for eligible patients. uHCC=unresectable hepatocellular carcinoma; TCI=Targeted Combined Immunotherapy; CE-MRI=Contrast-enhanced MR images.

**Figure 2:**
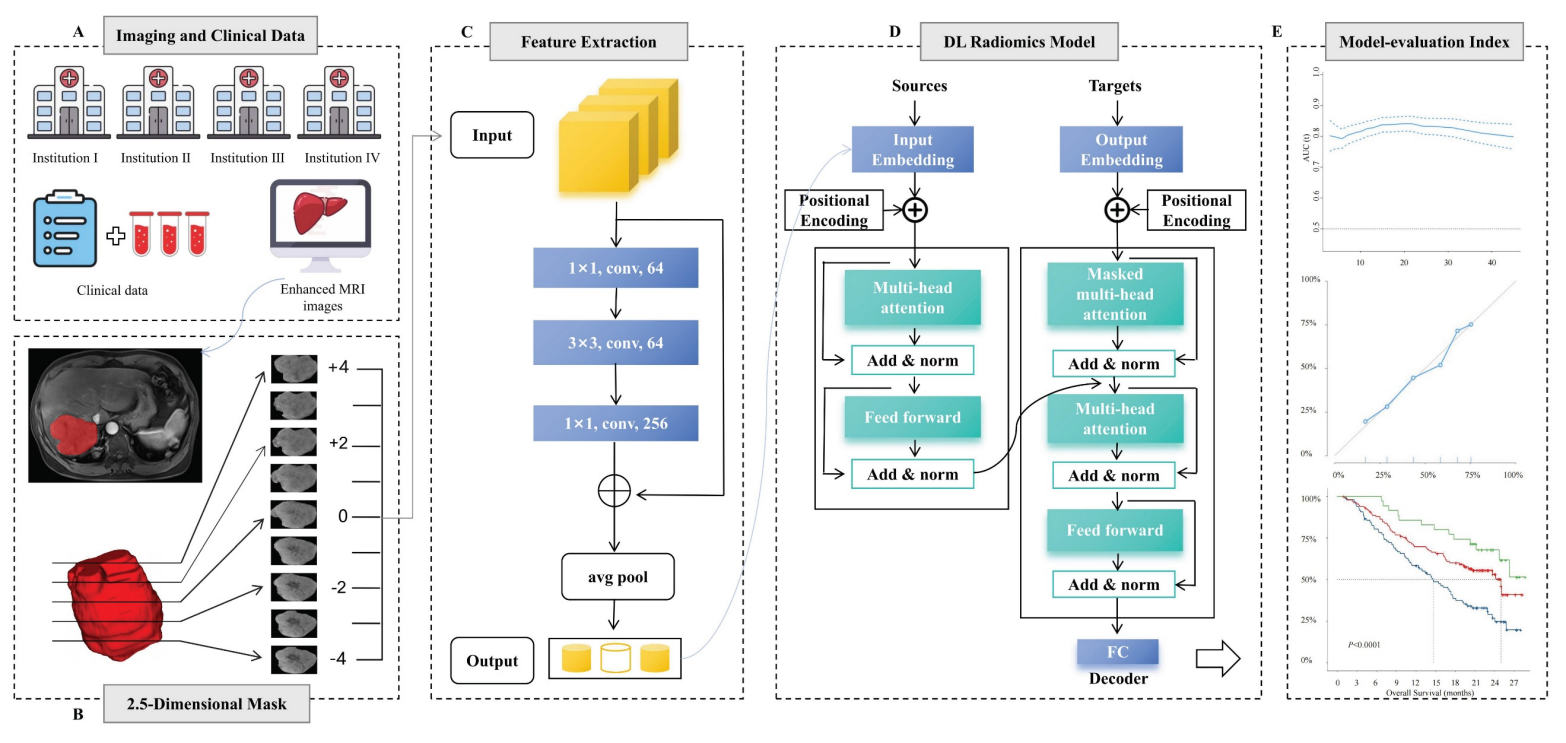
Workflow of developing deep learning models. **A** Clinical data and image acquisition. **B** 2.5D ROI segmentation, the largest tumor area was segmented into regions of interest (ROIs) for contrast-enhanced MRI, extracting the largest ROI layer along with its longitudinal layers from -4 to +4. **C** Deep learning feature extraction, utilize RESNET50 to extract features from 2.5D slices, selecting the deep learning features with the highest relevance through five-fold cross-validation. **D** Construct a predictive model based on the Transformer architecture. **E** Model performance verification, validate the efficacy of the model using Receiver Operating Characteristic (ROC) curves, Decision Curve Analysis (DCA) curves, and Kaplan-Meier curves.

A total of 264 eligible patients were included in the study, with 180 from Institution I comprising the training cohort, and 84 from other hospitals forming the independent validation cohort (Fig. 1). Of these, 206 (78%) received first-line therapy, 55 (20.8%) underwent second-line therapy, and 3 (1.2%) were treated with third-line therapy. In the training cohort, patients exhibited more adverse prognostic indicators (PS Score, Child-Pugh class, BCLC stage), a higher number of intrahepatic tumors, and more frequent portal vein invasion, all statistically significant (*P* < 0.05). No significant differences were observed between the training and validation cohorts in other baseline demographic and disease characteristics (Table 1). The ORR for the two cohorts were 26.1% (47/180) and 21.4% (18/84), respectively (*P* = 0.108).

In the univariate analysis of the entire cohort (Table S1), we examined the relationships between adverse tumor response and several clinical factors: Child-Pugh class B (OR 0.57; 95% CI: 0.26, 1.23), HBV infection (OR 0.72; 95% CI: 0.28, 1.85), portal hypertension (OR 0.77; 95% CI: 0.44, 1.34), and the presence of three or more tumors (OR 0.62; 95% CI: 0.33, 1.17). However, none of these relationships reached statistical significance (*P* > 0.05).

### Deep Learning Features Predicted the Objective Tumor Response

Based on expert evaluations using RECIST v1.1 criteria, patients were stratified into two groups: those with objective responses (CR/PR group) and those without (SD/PD group). Fig. S1. A, B illustrate the distribution of deep learning scores for patients in both the training and validation sets. Notably, patients in the CR and PR groups received high scores, whereas those in the SD and PD groups were assigned low scores. The analysis revealed significant differences between these groups. Importantly, the model demonstrated high concordance between its predictions of TCI responses in uHCC patients and the actual tumor response outcomes.

**Figure 3:**
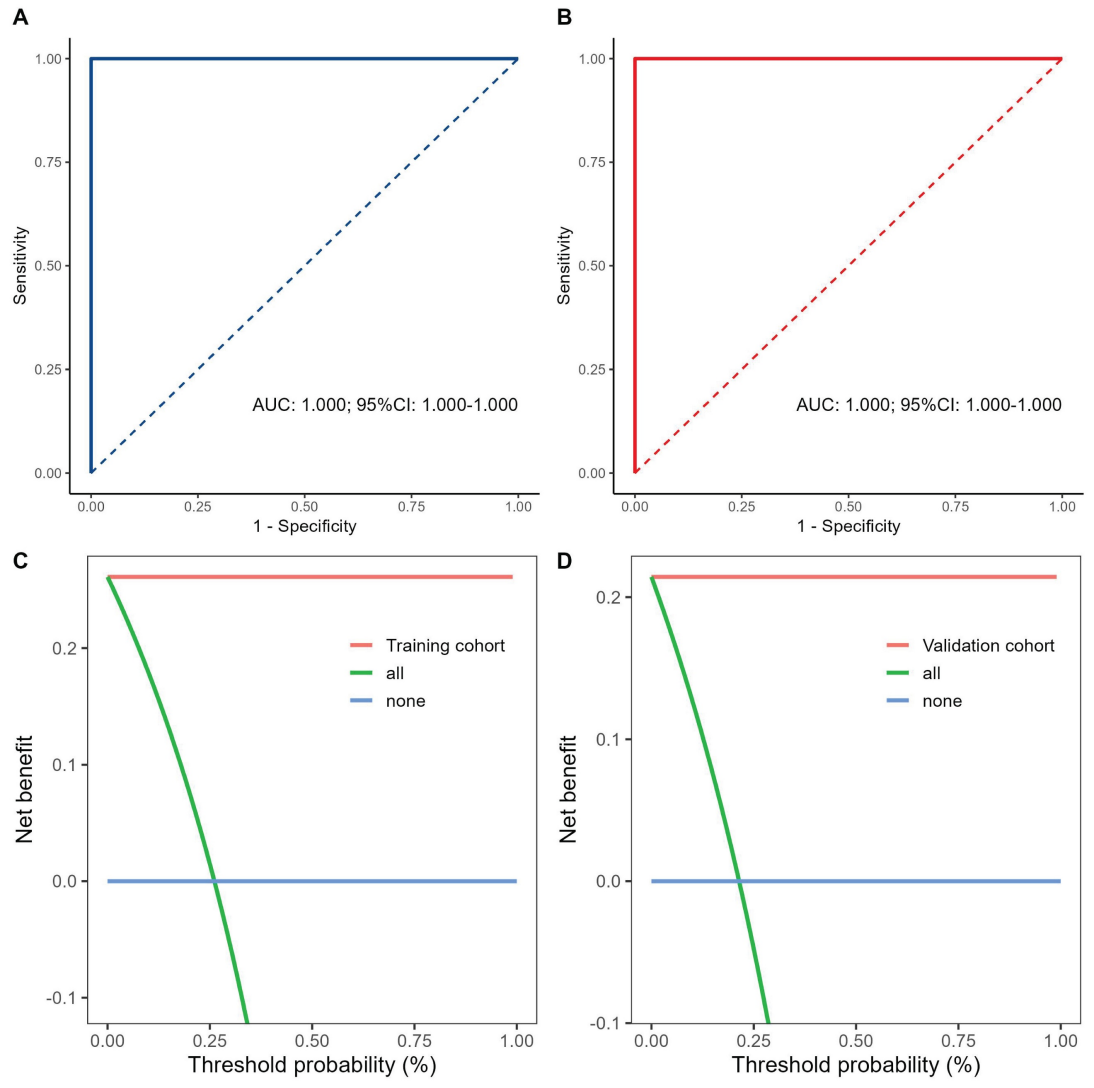
Performance and comparison of prediction models in the training and validation cohorts. **A, B** Analysis and comparison of the receiver operating characteristic curves for the predictive model in the training and validation sets.** C, D** Decision curve analysis of the predictive model in the training and validation cohorts.

Fig. 3. A and B illustrate the ROC curves for the TCI model in the training and validation cohorts, with both achieving AUC values of 1.000. Fig. 3. C and D show the DCA results, indicating that the proposed model yields superior net benefits within a reasonable range of threshold probabilities.

We performed random occlusions of series and layers in MRI scans to evaluate model performance sensitivity (Table 2). The occlusions of the PRE and AP series resulted in AUC decreases of 3.8% and 3.1%, respectively; however, these decreases were not statistically significant compared to the optimal AUC of 1.000 in the complete dataset. Moreover, occlusions in other layers minimally affected the AUC, demonstrating the model's robustness to partial data occlusion. This study confirms that the multi-instance learning and Transformer strategy effectively maintain high stability and performance, even with incomplete medical imaging data.

**Figure 4:**
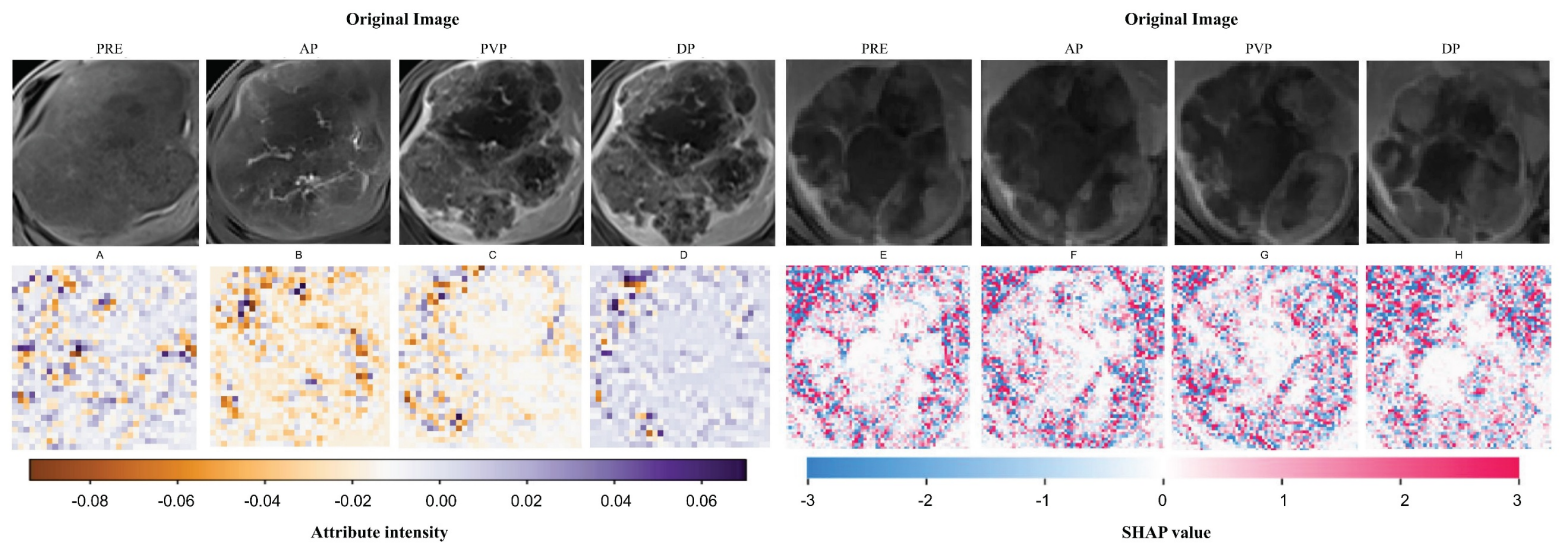
Visualization of feature mappings derived through ResNet50. **A-D** Visualization analysis of Patient A's full-sequence CE-MRI was conducted using the Grad-CAM algorithm. **E-H** Visualization analysis of Patient B's full-sequence CE-MRI was conducted using the SHAP algorithm. The full sequence is ordered as PRE, AP, PVP and DP.

To demonstrate the interpretability of the deep learning model, heatmaps were generated for two patients selected from the validation set using the Grad-CAM and SHAP algorithms. These heatmaps (Fig. 4) highlight the image regions that contribute most significantly to the network's decision-making process. Notably, hotspot regions are concentrated around the tumor, whereas areas of necrosis or liquefaction contribute less to the efficacy predictions. This aligns with the common understanding that regions of high malignancy are closely associated with prognosis.

### Radiomic Features Associated with OS and PFS

**Figure 5:**
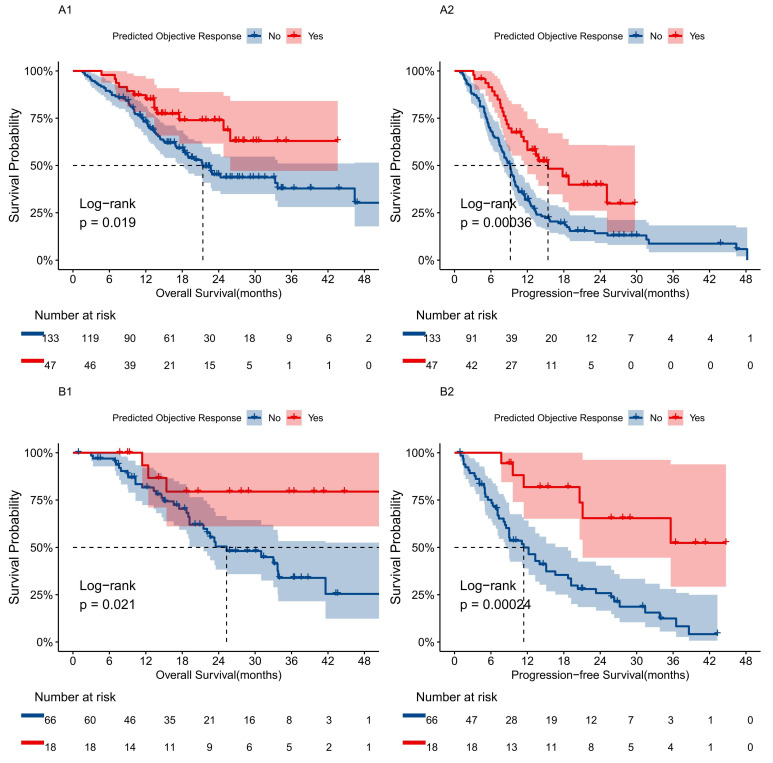
Survival prognosis analysis using a transformer model for uHCC patients receiving TCI treatment. **A1, B1** Kaplan-Meier curves for OS between predicted tumor response and non-response groups in the training and validation cohorts based on the transformer model. **A2, B2** Kaplan-Meier curves for PFS between predicted tumor response and non-response groups in the training and validation cohorts based on the transformer model.

We evaluated survival outcomes based on the presence of objective responses in patients, finding that those predicted to respond favorably demonstrated significantly better prognoses than non-responders. Kaplan-Meier analysis revealed that the mortality and disease progression rates for the predicted responder group were 27.7% and 53.2%, respectively, compared to 50.4% and 85.7% for the non-responder group (Fig. 5). The responder group showed significant survival advantages in OS (HR 0.50; 95% CI: 0.27, 0.90, *P*=0.0019) and PFS (HR 0.46; 95% CI: 0.30, 0.72, *P*=0.00036). Similar results were observed in the validation set.

### Assessment of the Model's Clinical Applicability

To assess the clinical applicability of the model, JPEG images of tumor target lesions captured via screenshots were used as input. Fig. S2. A and B compare the model's performance using screenshot images versus original image inputs, with AUC values of 0.929 (95% CI: 0.904, 0.947) and 1.000, respectively. The confusion matrix in Supplementary Fig. S2. C indicates that the model based on screenshot images is reasonably accurate in predicting tumor response, though a few misclassifications were observed.

## Discussion

In this study, we developed a Transformer model based on CE-MRI to predict long-term survival and treatment responses in uHCC patients undergoing TCI. Validated with a multicenter dataset, our model demonstrated a strong correlation between deep learning features and both OS and PFS following treatment. We visualized the prediction process using Grad-CAM and SHAP algorithms, and assessed the impact of using raw MR images versus screenshot images as inputs on model performance. To the best of our knowledge, this may be the first successful application of a multi-instance learning and Transformer strategy in the field of HCC MRI radiomics.

Although TCI protocols are effective in some uHCC patients, less than 30% experience benefits 6-8. Previous studies have identified various biomarkers related to immunotherapy prognosis, including tumor characteristics (PD-1/PD-L1 expression 23, TMB 24), tumor microenvironment (CD3/CD8 tumor-infiltrating lymphocytes 25, dMMR/MSI 26), peripheral blood markers (NLR/PLR 27, ctDNA 28 ALFP score 29), and gut microbiota 30. Despite this, the high heterogeneity of HCC and complex immune response mechanisms 31-33 challenge the practicality and efficacy of these biomarkers.

Radiomics-based approaches have shown promising potential, including models proposed by Xu et al. 34, Wei et al. 35, and Shen et al. 36. Notably, the first two studies reported AUC values of 0.820 and 0.882, respectively, for predicting treatment response. A recent multi-parametric MRI fusion model proposed by Kang et al. 37 achieved an AUC of 0.869 for similar tasks. Additionally, a recent study 38 using a machine learning classifier to predict the effects of LPI treatment, achieved an AUC of 0.893, further validating the applicability of radiomics in predicting immunotherapy outcomes. In comparison with these studies, the significant increase in AUC observed in this study can be attributed to the use of more advanced strategies. The combination of MIL and Transformer models enables dynamic establishment of image feature correlations across anatomical regions, thereby more effectively integrating complementary information from multi-sequence MRI. In a multicenter study 39 of over 13,000 individuals assessing microsatellite instability in colorectal cancer HE-WSI, researchers extracted essential features from each patch and synthesized them into WSI labels using a five-fold cross-validation Transformer model. The findings indicated that both the negative and positive predictive values of the model achieved 0.99, reaching clinical-grade accuracy. This study confirms the efficacy of this advanced methodological integration in pathological research.

Our dataset included patients undergoing various lines of combination immunotherapy, enhancing the clinical generalizability of findings. Although traditional clinical-pathological features (such as AFP levels, extrahepatic metastasis, and maximum tumor diameter) showed no significant correlation with treatment response (*P* >0.05), the radiomics-based predictive model maintained excellent stability in validation across three independent medical centers, confirming its robustness against equipment variations. In this study, there was no statistically significant difference in predictive performance between screenshot JPG data and original DICOM images (AUC: 0.89 vs. 0.91, *P* =0.12), consistent with the findings of Sedlaczek et al. 40, indicating that screenshot-based analysis can reduce data coordination costs and streamline clinical translation pathways. Through visual analysis, we explored potential connections between deep learning features, tumor heterogeneity, and the immune microenvironment. However, these associations need further validation through additional genomic and histopathological studies.

This study has several limitations. First, as a multicenter study, differences in MRI scanner performance and imaging techniques, despite efforts to standardize images, may have influenced feature extraction. Second, subjectivity involved in manually delineating ROIs and the potential loss of crucial information related to the tumor microenvironment represent additional limitations. In future studies, we plan to expand the cohort to five centers and apply an automatic segmentation algorithm based on 3D U-Net 41 to systematically assess quantitative feature variations in the tumor/liver parenchyma. Third, the retrospective nature of the study and the relatively limited sample size also introduce potential selection bias. Future work should focus on larger, prospective studies to optimize and refine the model.

This study preliminarily validates the efficacy of combining multi-instance learning with the Transformer method in radiomics, demonstrating that the model, which utilizes screenshot data, achieves high accuracy in predicting treatment outcomes. Currently, the use of radiomics technology for predicting HCC immunotherapy outcomes is confined to small-scale exploratory studies. Future research will necessitate larger sample sizes and more extensive data from multiple centers to further confirm the reliability of these findings. Specifically, robust validation of the stability of MRI screenshot data could significantly facilitate clinical applications.

## Supplementary Material

Supplementary figures and table.

## Figures and Tables

**Table 1:** Baseline characteristics of patients in the training cohort and validation cohort

Variables	Training cohort (N=180)	Validation cohort (N=84)	*P* value
Age(yr)	55.5 ± 12.3	54.2 ± 12.0	0.411
Sex			0.728
Female	27 (15.0%)	14 (16.7%)	
Male	153 (85.0%)	70 (83.3%)	
HBV			0.209
No	13 (7.2%)	10 (11.9%)	
Yes	167 (92.8%)	74 (88.1%)	
Child-Pugh class			0.003
A	135 (75.0%)	76 (90.5%)	
B	45 (25.0%)	8 (9.5%)	
Log AFP	2.1 ± 1.3	2.4 ± 1.3	0.221
Portal hypertension			0.002
No	79 (43.9%)	54 (64.3%)	
Yes	101 (56.1%)	30 (35.7%)	
PVTT			<0.001
No	76 (42.2%)	57 (67.9%)	
Yes	104 (57.8%)	27 (32.1%)	
PS Score			<0.001
0	41 (22.8%)	43 (51.2%)	
1	104 (57.8%)	40 (47.6%)	
2	35 (19.4%)	1 (1.2%)	
Largest tumor size(cm)	8.1 ± 4.6	7.7 ± 4.6	0.513
Tumor number			<0.001
1	56 (31.1%)	33 (39.3%)	
2	43 (23.9%)	6 (7.1%)	
3	32 (17.8%)	5 (6.0%)	
>3	49 (27.2%)	40 (47.6%)	
Extrahepatic metastasis			0.779
No	119 (66.1%)	57 (67.9%)	
Yes	61 (33.9%)	27 (32.1%)	
BCLC stage			0.003
A	8 (4.4%)	14 (16.7%)	
B	32 (17.8%)	15 (17.9%)	
C	140 (77.8%)	55 (65.5%)	
Response			0.108
CR	0 (0.0%)	1 (1.2%)	
PR	47 (26.1%)	17 (20.2%)	
SD	44 (24.4%)	30 (35.7%)	
PD	89 (49.4%)	36 (42.9%)	
Treatment line			0.205
1	145 (80.6%)	61 (72.6%)	
2	34 (18.9%)	21 (25.0%)	
3	1 (0.6%)	2 (2.4%)	

Differences are compared using the chi-square test (or Fisher's exact test) for categorical measures and Kruskal-Wallis test for continuous measures. AFP=α-fetoprotein; PVTT=portal vein tumor thrombus; PS=Eastern Cooperative Oncology Group performance status; BCLC=Barcelona Clinic Liver Cancer.

**Table 2:** The change of AUC after random occlusion series or layers of MRI

Cohort	Model	AUC	95%CI	change
Series	Baseline	1.000	(1.000, 1.000)	0.0%
	-PRE	0.962	(0.922, 0.992)	-3.8%
	-AP	0.969	(0.939, 0.993)	-3.1%
	-PVP	0.990	(0.971, 1.000)	-1.0%
	-DP	1.000	(1.000, 1.000)	0.0%
Layers	-0	0.992	(0.975, 1.000)	-0.8%
	±2	1.000	(1.000, 1.000)	0.0%
	±4	0.992	(0.975, 1.000)	-0.8%
