# Supplementary material for: Pretreatment Multi-sequence Contrast-Enhanced MRI to Predict Response to Immunotherapy in Unresectable Hepatocellular Carcinoma Using Transformer: A Multicenter Study
Source: J Cancer. 2025 Jun 12;16(8):2663–72. doi: 10.7150/jca.111026 (PMC12170994; doi:10.7150/jca.111026)
Supplement: Supplementary file 1 — Supplementary figures and table. [file jcav16p2663s1.pdf]

Table S1. Univariate logistic regression analyses of clinicopathologic features in the training cohort between patients with and without an objective response

Features	Statistics	Odds ratio	95% CI	*P* value
Age				
≤55	139 (52.65%)	1		
>55	125 (47.35%)	1.2	0.68-2.10	0.5249
BCLC stage				
A	22 (8.33%)	1		
B	47 (17.80%)	0.7	0.20-2.44	0.5733
C	195 (73.86%)	1.24		0.6911
Child-Pugh class				
A	211 (79.92%)	1		
B	53 (20.08%)	0.57	0.26-1.23	0.1526
Sex				
Female	41 (15.53%)	1		
Male	223 (84.47%)	1.42	0.62-3.24	0.4104
HBV				
No	23 (8.71%)	1		
Yes	241 (91.29%)	0.72	0.28-1.85	0.4997
Portal hypertension				
No	133 (50.38%)	1		
Yes	131 (49.62%)	0.77	0.44-1.34	0.3532
Largest tumor size	7.95 ± 4.57	1.05	0.98-1.11	0.1437
Tumor number				
<3	175 (66.29%)	1		
≥3	89 (33.71%)	0.62	0.33-1.17	0.1396
Extrahepatic metastasis				
No	176 (66.67%)	1		
Yes	88 (33.33%)	1.13	0.63-2.03	0.6862
PVTT				
No	133 (50.38%)	1		
Yes	131 (49.62%)	1.74	0.99-3.08	0.0553

BCLC=Barcelona Clinic Liver Cancer; PVTT=portal vein tumor thrombus.

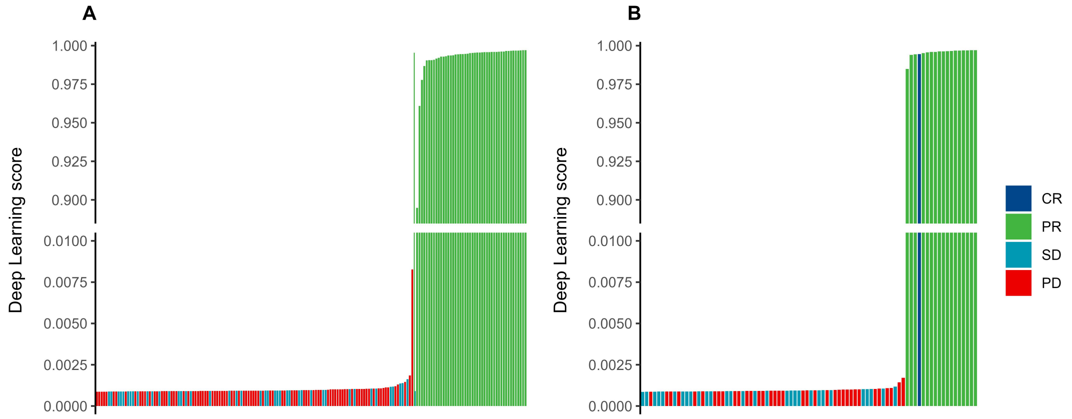

Fig. S1. Deep learning scores for each patient in the training and validation cohorts.

A Represents the training set. **B** Represents the validation set. CR=Complete response; PR=Partial response; SD=Stable disease; PD=Progressive disease.

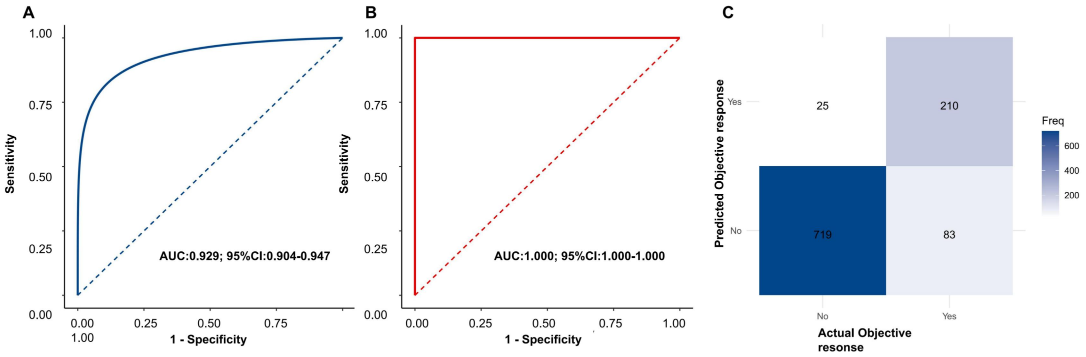

Fig. S2. Sample-level AUC curves and confusion matrices.

A ROC curve of the ResNet50 model based on screenshot images in the validation set. **B** ROC curve of the model using original image inputs. **C** Confusion matrix for the model with screenshot image inputs.
